# Guiding first-line treatment decisions in advanced urothelial carcinoma: a global survey

**DOI:** 10.1093/oncolo/oyaf333

**Published:** 2025-09-06

**Authors:** Enrique Grande, Joaquim Bellmunt, Syed A Hussain, Mubarak M Al Mansour, Aristotle Bamias, Philippe Barthélémy, David J Benjamin, Normand Blais, Maria T Bourlon, Daniel Castellano, Pongwut Danchaivijitr, Mauricio Fernandez Lazzaro, Patrizia Giannatempo, Félix Guerrero-Ramos, Roberto Iacovelli, Philipp Ivanyi, Eun Hee Jung, Ravindran Kanesvaran, Ray Manneh, Joana C Marinho, Nobuaki Matsubara, Axel S Merseburger, Deborah Mukherji, Chandler H Park, Ben Tran, Karine Martins da Trindade, Yüksel Ürün, Ashish M Kamat, Alison J Birtle

**Affiliations:** Department of Medical Oncology, MD Anderson Cancer Center Madrid and Universidad Francisco de Vitoria/Facultad de Medicina, Madrid 28033, Spain; Facultad de Medicina, Universidad Francisco de Vitoria, Pozuelo de Alarcón 28223, Spain; Department of Internal Medicine, Dana Farber Cancer Institute, Boston, MA 02215, United States; Division of Clinical Medicine, University of Sheffield, School of Medicine and Population Health, Sheffield S10 2RX, United Kingdom; Princess Noorah Oncology Center, Oncology Department, King Abdulaziz Medical City Ministry of National Guard Health Affairs, Jeddah 22384, Kingdom of Saudi Arabia; 2nd Propaedeutic Department of Internal Medicine, National & Kapodistrian University of Athens, Chaidari 12462, Greece; Medical Oncology Department, University Hospital Strasbourg, Strasbourg 67000, France; Medical Oncology, Hoag Family Cancer Institute, Newport Beach, CA 92663, United States; Medicine, CHUM, Montreal H2X 0C1, Canada; Hemato-Oncology Department, Urologic Oncology Clinic, Instituto Nacional de Ciencias Médicas y Nutrición Salvador Zubirán, Mexico City 14080, Mexico; Medical Oncology, Hospital Universitario 12 de Octubre, Madrid 28041, Spain; Department of Medicine, Faculty of Medicine, Siriraj Hospital Mahidol University, Bangkok 10700, Thailand; Department of Uro-Oncology, Fundacion COIR, Mendoza, M5500 Argentina; Department of Genitourinary Oncology, Fondazione IRCCS Istituto Nazionale dei Tumori, Milan 20133, Italy; Department of Urology, Hospital Universitario 12 de Octubre, Madrid 28041, Spain; Department of Medical and Surgical Sciences, Medical Oncology Unit, Catholic University of Sacro Cure—Fondazione Policlinico Universitario A. Gemelli IRCCS, Rome 00168, Italy; Department of Hematology, Hemostasis, Oncology and Stem Cell Transplantation, Hannover Medical School, Hannover 30625, Germany; Department of Internal Medicine, Division of Hematology and Medical Oncology, Seoul National University Bundang Hospital, Seongnam-si 13620, Republic of Korea; Division of Medical Oncology, National Cancer Centre Singapore, Singapore 168583, Singapore; Department of Clinical Oncology, Sociedad de Oncología y Hematología del Cesar, Valledupar, Colombia; Department of Medical Oncology, Gaia Espinho Local Health Unit, Vila Nova de Gaia 4434-502, Portugal; Department of Medical Oncology, National Cancer Center Hospital East, Kashiwa 277-8577, Japan; Department of Urology, University Hospital Schleswig-Holstein, University Lübeck, Lübeck 23562, Germany; Department of Hematology Oncology, Clemenceau Medical Center Dubai, Dubai, United Arab Emirates; School of Medicine, Norton Cancer Institute/University of Louisville, Louisville, KY 40202, United States; Department of Medical Oncology, Peter MacCallum Cancer Centre, Melbourne 8006, Australia; Oncologia D’Or, Fortaleza 60170-170, Brazil; Department of Medical Oncology, Ankara University School of Medicine, Ankara 06230, Turkey; Department of Urology, The University of Texas MD Anderson Cancer Center, Houston, TX 77030, United States; Department of Oncology, Rosemere Cancer Centre, Lancs Teaching Hospitals, University of Manchester, University of Central Lancashire, Preston PR2 9HT, United Kingdom

**Keywords:** urothelial carcinoma, enfortumab vedotin, systemic treatment, individualization, criteria

## Abstract

**Background:**

Enfortumab vedotin combined with pembrolizumab (EV-P) has become the new standard first-line therapy for patients with advanced urothelial carcinoma (aUC), based on its superior efficacy over platinum-based chemotherapy. As this regimen is increasingly adopted in routine care, treatment decisions may often occur in sites without dedicated genitourinary oncology expertise. This global survey aimed to explore how physicians perceive clinical factors that may influence the safe and effective use of EV-P in daily practice.

**Material and methods:**

A panel of international physicians with experience in treating patients with genitourinary cancers developed a 17-question survey addressing practice settings, experience in managing aUC, and clinical considerations relevant to the use of EV-P. The participants were recruited through a network-based convenience sampling method. The responses were descriptively analyzed.

**Results:**

A total of 201 genitourinary physicians from 32 countries completed the questionnaire. The most frequently cited potential absolute contraindications were sensory or motor neuropathy grade ≥2 (64.2%), ECOG-PS ≥3 (59.2%), and non–urothelial component >50% of the tissue sample (59.2%). Other notable concerns included severe corneal/retinal abnormalities, HbA1c >11%, severe skin comorbidities, liver impairment grade ≥2, and dialysis dependence.

**Conclusions and relevance:**

This survey provides practical insights into real-world physician perspectives on patient selection for EV-P. The findings highlight the need for guidance to support personalized risk assessment, facilitate early identification of patients who may require enhanced monitoring, and optimize safe integration of EV-P into clinical practice.

Implications for practice This is the first global survey to assess real-world perceptions of clinical factors conditioning the selection of patients with advanced urothelial carcinoma for enfortumab vedotin (EV-P) eligibility in daily practice. The survey identified key clinical factors, such as neuropathy, ECOG Performance Status ≥3, histology variants, and uncontrolled diabetes, as commonly perceived barriers to EV-P use, many of which align with the exclusion criteria in the EV-302 trial. These insights provide a foundation for developing consensus-based recommendations, enhancing patient selection, and optimizing the safe implementation of EV-P in routine clinical practice.

## Introduction

The unprecedented efficacy results of enfortumab vedotin-pembrolizumab (EV-P) compared with standard platinum-based chemotherapy in untreated patients with advanced urothelial carcinoma in the EV-302 trial^1^ have set this combination as the new standard first-line therapy if available and not contraindicated.^2–4^ The combination of EV-P is associated with a distinct safety profile, notably characterized by hyperglycemia, peripheral neuropathy, and skin toxicity in addition to immune-related side effects.^1^^,^^5^ These adverse events, when layered over common comorbidities observed in patients diagnosed with advanced urothelial carcinoma, may potentially limit the broader use of this regimen in clinical practice. There are no formally stablished contraindications for EV-P, which means that physicians must use their own clinical judgment to select patients for this regimen in daily practice.^6^ In most countries, patients with advanced urothelial carcinoma are frequently managed by medical oncologists working in community-based hospitals who are not subspecialized in genitourinary cancers, with a mean of only 2 patients with advanced bladder cancer treated per physician per year.^7^ Community-based physicians may not always have sufficient experience to accurately identify patients at higher risk of developing severe treatment-related toxicities.^8^

To address this gap, following the inclusion of EV-P in international treatment guidelines,^2–4^ we conducted a global survey to evaluate how genitourinary physicians worldwide believe diverse demographic and clinical factors might guide its safe and effective use in daily practice.

## Materials and methods

This survey was conducted between February 2025 and March 2025. A panel of internationally recognized experts in genitourinary tumors (the byline authors) developed a questionnaire composed of 17 questions (Supplementary Material): 5 questions concerning the practice settings and experiences of the respondents in managing advanced urothelial carcinoma (Q1-Q5), 2 questions concerning previous experiences with EV-P (Q6-Q7), 1 question concerning the usefulness of having consensus criteria that could guide the assessment of unsuitability for receiving EV-P (Q8), 4 questions concerning the clinical factors used in practice for determining the use of EV-P (Q9-Q12), 1 question concerning the preferred treatment option for those patients who are ineligible for EV-P (Q13), 3 questions concerning the use of biomarkers (Q14-Q15) or histology (Q16) for the use of the combination treatment, and a final question concerning whether the cost of treatment with EV-P was a significant barrier to the use of this combination treatment (Q17).

The participants (expand with findings coming from Q3) were identified using a network-based convenience sampling technique.^9^ Each byline author nominated experts, who in turn referred to additional participants. These individuals were contacted via e-mail with a brief explanation of the project and a link to the online survey; the non–respondents received 1 reminder 5 days after the initial contact. This survey was designed as an exploratory, hypothesis-generating project. Responses were analyzed descriptively, with the results presented as absolute and relative frequencies. Given the non–probabilistic, convenience sampling approach, no inferential statistical testing or calculation of confidence intervals was performed, as these would not be representative of the underlying population. Exploratory subgroup analyses by region, years of experience, and practice setting were considered, but no consistent or interpretable patterns emerged, largely due to small numbers within subgroups; therefore, detailed subgroup findings are not reported.

## Results

**Figure 1.**
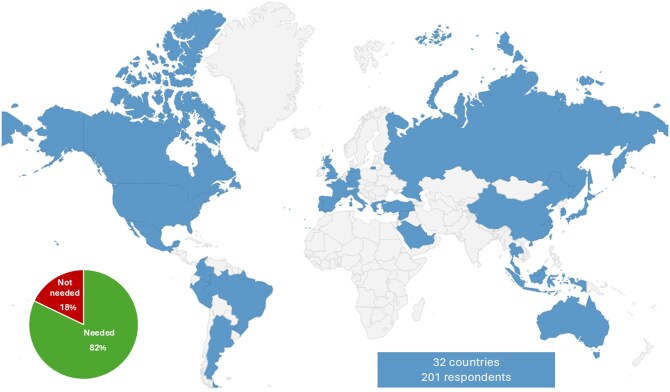
Global map showing the diverse origins of survey respondents and the need to identify patients in clinical practice for whom enfortumab vedotin and pembrolizumab may pose excessive risk for toxicity.

Overall, 626 physicians treating patients with advanced urothelial cancer from 32 countries were invited to participate in this survey, and 201 (32.1%) completed the questionnaire. The respondents originated from Europe (*n* = 72); the Middle East and Asia (*n* = 48); Latin America and the Caribbean (*n* = 46); Canada and the United States (*n* = 27); and Australia (*n* = 4) (Figure 1). The profiles of the respondents are summarized in Table 1. Percentages are reported descriptively without confidence intervals, in line with the exploratory and non–random nature of the survey.

**Table 1.** Characteristics of the respondents.

Characteristic	*n*	%
Working site (Question 1)		
** Academic center with ≥1000 beds**	52	25.9
** Academic center with 500-999 beds**	52	25.9
** Academic center with 200-499 beds**	54	26.9
** Academic center with <200 beds**	23	11.4
** Community center**	20	10.0
**Specialty (Question 2)**		
** Medical/clinical oncologist**	183	91.0
** Urologist**	18	9.0
**Daily practice (type of tumors) (Question 3)**		
** I am treating patients with genitourinary tumors only**	96	47.8
** I am treating patients with genitourinary tumors and up to 2 other solid tumor types**	78	38.8
** I treat solid tumor types including genitourinary tumors**	24	11.9
** I am treating urothelial carcinoma only**	3	1.5
**Daily practice (experience with advanced urothelial carcinoma) (Question 4)**		
** >50 advanced/metastatic UC patients per year**	79	39.3
** >30 advanced/metastatic UC patients per year**	39	19.4
** 21-30 advanced/metastatic UC patients per year**	38	18.9
** 11-20 advanced/metastatic UC patients per year**	29	14.4
** 6-10 advanced/metastatic UC patients per year**	12	6.0
** 1-5 advanced/metastatic UC patients per year**	4	2.0

Abbreviation: UC, urothelial carcinoma.

**Figure 2.**
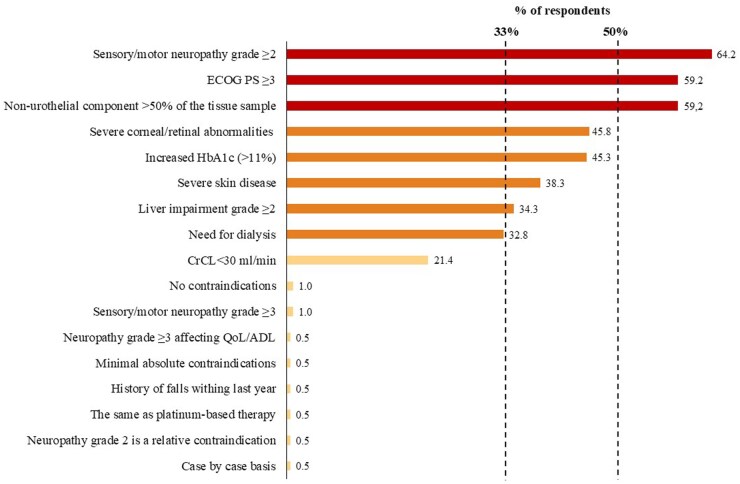
Absolute contraindications for enfortumab vedotin and pembrolizumab as A first-line treatment for patients with advanced urothelial carcinoma in daily practice. Abbreviations: ADL, activities of daily life; CrCl, creatinine clearance; ECOG PS, Eastern Cooperative Oncology Group Performance Status; QoL, quality of life.

Most of the respondents (*n* = 165, 82.1%) considered that oncologists treating fewer than 5 patients with advanced urothelial carcinoma per year would benefit from having consensus guidance to optimize patient suitability for EV-P. To select patients for EV-P, formal Galsky’s^10^ and modified Gupta’s criteria^11^ were used in daily practice by 88 (43.8%) and 58 (28.9%) of respondents, respectively. The aspects most frequently considered by respondents to evaluate the suitability of EV-P are shown in Supplementary Figure S1. The patient’s conditions or clinical factors commonly felt to be absolute contraindications for more than 50% of the respondents were the presence of sensory or motor neuropathy grade ≥2 (*n* = 129, 64.2%), an Eastern Cooperative Oncology Group performance status (ECOG PS) of  ≥3 (*n* = 119, 59.2%), and a non–urothelial component higher than 50% of the tissue sample (*n* = 119, 59.2%). Other clinical factors that were highlighted by at least one-third of the respondents as absolute contraindications for the use of EV-P included severe corneal or retinal abnormalities (*n* = 92, 45.8%), an increased HbA1c level of >11% (*n* = 91, 45.3%), severe skin comorbidities (*n* = 77, 38.3%), liver impairment grade ≥2 (*n* = 69, 34.3%), and the need for dialysis (*n* = 66, 32.8%) (Figure 2). Two (1.0%) respondents considered that there were no factors that could be determined as absolute contraindications. One hundred forty respondents (69.7%) did not consider age a key factor that could affect the suitability of EV-P.

For patients who were considered to be not candidate for EV-P, the preferred treatment option was platinum-based chemotherapy followed by maintenance avelumab treatment (*n* = 107, 53.2%), the combination of nivolumab-gemcitabine-cisplatin (*n* = 27, 13.4%) assuming patients were eligible for cisplatin, carboplatin-gemcitabine only (*n* = 23, 11.4%), and finally single agent use of PD-1/L1 regimen (*n* = 21, 10.4%). For 119 (59.2%) participants, cost was a significant barrier to using EV-P. All the questions and responses are available in Table 1 and supplementary Tables S1-S13.

## Discussion

The combination of EV-P has emerged as the mainstay of systemic treatment for advanced urothelial carcinoma, and it is poised to be a key therapeutic option even in the earlier stages of the disease. Although its use in clinical practice is expected to become widespread once cost-related access barriers are overcome, its distinct safety profile demands careful consideration. The diverse clinical profiles and comorbidities of patients with advanced urothelial carcinoma underscore the need for individualized treatment strategies rather than a one-size-fits-all approach. The potential for severe and, in some cases life-threatening toxicities highlights the need for meticulous patient selection to ensure optimal outcomes.

Most of the clinical parameters identified in the survey are related to complications arising from poorly controlled or longstanding diabetes mellitus, such as diabetic neuropathy, retinopathy, and nephropathy. In this context, an elevated HbA1c level (>11%) emerges as a particularly relevant marker for a fatal prognosis. According to the American Diabetes Association guidelines, glycated hemoglobin is a well-established indicator of chronic glycemic control and is strongly associated with the development of the aforementioned complications and adverse outcomes, including increased mortality and cardiovascular events.^12^^,^^13^

Pre–habilitation strategies to prevent the appearance and severity of toxicities have demonstrated benefits in other malignancies, where the optimization of modifiable clinical parameters before treatment has improved both patient eligibility and outcomes.^14^ In the setting of EV-P, factors such as uncontrolled hyperglycemia can be pre–emptively managed with widely available interventions.^14^ However, the aggressive nature of advanced urothelial carcinoma often limits the window for such pre–habilitation. This gap between theoretical benefits and clinical feasibility underscores the need for streamlined, rapid-acting strategies that can allow immediate access to treatments without delaying oncologic intervention. Conversely, baseline factors such as pre–existing peripheral neuropathy, an ECOG performance status score ≥3, severe retinopathy, dialysis dependence, or non–obstructive liver impairment remain difficult to optimize and may limit the real-world use of EV-P.

Taken together, our results support the notion that the vast majority of patients with metastatic urothelial carcinoma are potential candidates for EV-P therapy, consistent with the unprecedented efficacy reported in EV-302. While clinical factors remain critical for assessing suitability and safety, they may not fully capture the complexity of treatment decision-making in the future. Emerging biomarkers—such as genomic alterations, transcriptomic profiles, or immunologic signatures—may ultimately provide more precise tools to guide the selection of patients most likely to benefit from EV-P while minimizing unnecessary toxicity. Integrating biomarker-based approaches with clinical judgement will be essential to advance toward a more personalized and evidence-driven implementation of EV-P in daily practice.

Beyond patient selection, the optimal management of patients receiving EV-P is a central issue in clinical practice. The success of this regimen depends not only on identifying appropriate candidates but also on implementing proactive strategies for early recognition and management of toxicities such as hyperglycemia, neuropathy, or dermatologic complications. Close monitoring, patient education, and multidisciplinary supportive care are essential to maintain treatment intensity, minimize discontinuations, and optimize outcomes. Future consensus efforts and real-world studies should therefore address both the clinical factors guiding eligibility and the strategies required for optimal management during therapy.

This study has several limitations. First, the referral-based sampling method used to recruit survey participants may have introduced a selection bias, potentially overrepresenting academic specialists and underrepresenting community-based oncologists (where more than 50% of advanced urothelial cancer patients are treated according to the respondents). Indeed, 181 respondents (90%) reported practicing in academic institutions, whereas only 20 respondents (10%) came from community centers. This imbalance suggests that the perspectives captured here may be more reflective of academically oriented or higher-volume practices and therefore may not fully represent the views and challenges faced by community-based oncologists, where a substantial proportion of patients with advanced urothelial carcinoma are managed. Second, the geographical distribution of respondents was not uniform, with certain regions such as North America (*n* = 27) and Australia (*n* = 4) being underrepresented. This is noteworthy, as these regions were among the first to obtain access to EV-P both in clinical trials and in routine practice, and therefore physicians from these areas may have had greater direct experience with the regimen. At the same time, one of the key strengths of this survey is its global scope, encompassing responses from 32 countries with markedly different healthcare systems, levels of resources, and cultural contexts. Since the majority of patients with advanced urothelial carcinoma are managed in regions outside of North America and Western Europe, we consider this diversity a unique asset, enabling the survey to capture not only heterogeneous levels of experience with EV-P but also varying perceptions of safety and toxicity management across different ethnic and genetic backgrounds. Third, the survey reflects physician perspectives at a single time point and may not capture evolving clinical experience or the influence of newly emerging data. Fourth, not all respondents had direct or extensive experience with EV-P in routine clinical practice (43.3% of the respondents had treated fewer than 10 patients with EV-P at the time of responding). Therefore, the survey might not specifically capture respondents’ direct experience with the EV-P combination. At the time the survey was conducted, access to EV-P was restricted in several countries due to reimbursement and regulatory factors, and many physicians may therefore have had minimal or no hands-on exposure to the regimen outside of clinical trial settings. As such, the findings should be interpreted as reflecting physician perceptions and anticipated thresholds for contraindications, rather than systematically accumulated real-world experience with EV-P in daily practice. Fifth, although exploratory subgroup analyses were performed (eg, by geographic region, years of clinical experience, and practice setting), no consistent differences were identified, and the small sample sizes within certain categories precluded meaningful interpretation. Finally, the absence of patient-level data limits the ability to correlate the survey findings with real-world outcomes.

Controversy exists on the need to define absolute restrictive criteria for EV-P use.^6^ Our survey of 201 genitourinary physicians underscores the critical importance of clinical vigilance and the need for practical guidance to assist non–expert clinicians in identifying patients at an increased risk of treatment-related toxicities. These findings are not intended to limit access to EV-P therapy but rather to support individualized decision-making, enabling clinicians to identify patients for whom alternative approaches or enhanced monitoring may be more appropriate.

Our study did not explore optimal therapies based on specific clinical scenarios. For instance, cisplatin-based chemotherapy would likely not be favored in patients with significant renal dysfunction or pre–existing neuropathy, and treating patients with ECOG PS ≥ 3 remains challenging regardless of the regimen chosen. In clinical practice, some physicians may consider empirical dose modifications of EV, such as initiating treatment with a reduced dose or omitting the Day 8 administration, in an effort to improve tolerability in frail patients or those with borderline fitness. However, these strategies remain unsupported by prospective clinical data, and their impact on efficacy cannot be directly compared with other alternative regimens such as carboplatin plus gemcitabine or single-agent PD-1/PD-L1 inhibitors. It is important to emphasize that such empirical practices should not be considered evidence-based, but rather anecdotal adaptations reflecting the heterogeneity of real-world clinical scenarios. It is also noteworthy that several of the clinical factors identified as potential contraindications for EV-P overlap with those that preclude the safe use of platinum-based chemotherapy, such as severe neuropathy, renal dysfunction, poor performance status (ECOG PS > 2), or uncontrolled diabetes. This overlap underscores that many patients considered unsuitable for EV-P are, in fact, broadly unfit for any multi–agent systemic regimen, rather than uniquely ineligible for this specific combination. The recent EAU guidelines^4^ highlight this point clearly, emphasizing the need for individualized treatment approaches for such patients, which often include single-agent immunotherapy or best supportive care. Furthermore, some of the contraindications identified by respondents, such as significant liver impairment, severe ocular abnormalities, or extensive dermatologic comorbidities, are also relevant for checkpoint inhibitor therapy in general. These factors may preclude not only the use of EV-P but also the administration of single-agent PD-1/PD-L1 inhibitors in the first-line, maintenance, or later-line setting. This observation suggests that part of the perceived contraindication spectrum reflects the broader safety profile of immunotherapy agents, rather than being exclusive to the antibody-drug conjugate component of EV-P.

This underscores the urgent need for real-world evidence studies involving a significant number of patients treated with EV-P to better understand the effectiveness, safety, and feasibility of this regimen across a broader clinical spectrum than that represented in clinical trials. Such data could inform future treatment adaptations and provide practical guidance for tailoring therapy in everyday oncology settings.

This survey aimed to capture physicians’ perceptions and assumptions in the context of a lack of prospective or real-world data, particularly for patients with advanced urothelial carcinoma who are commonly excluded from clinical trials due to the presence of factors highlighted in this survey. These findings should therefore be interpreted as expert-derived perceptions and self-reported clinical thresholds, rather than as evidence of actual treatment patterns or patient outcomes in daily practice.

Our survey also provides an important opportunity for future research aimed at designing clinical trials to address these unmet needs, either by testing novel agents that target pathways beyond nectin-4 or different payloads or by developing different doses and treatment schedule modifications of EV-P.

## Conclusions

In the present survey, the following clinical factors were identified by at least one-third of the respondents as potential absolute contraindications to EV-P: the presence of sensory or motor neuropathy grade ≥2, an ECOG PS of ≥ 3, non–urothelial component higher than 50% of the tissue sample, severe corneal or retinal abnormalities, an increased HbA1c level of >11%, severe skin comorbidities, liver impairment grade ≥2, and the need for dialysis. It is important to highlight that all of these factors were exclusion criteria in the EV-302 trial. In the absence of validated biomarkers, head-to-head comparison in diverse unexplored settings and evidence, first-line systemic treatment selection for advanced urothelial carcinoma continues to depend on clinical judgement and patient preferences, guided by the best available physician perception/assumptions linked to the personal experience. This survey offers practical and guideline-informing insights that may help physicians identify advanced urothelial cancer patients for whom EV-P may pose an excessive risk to develop severe toxicities and, therefore, to apply individualized proactive monitoring and supportive care to mitigate toxicity without compromising timely cancer treatment. Ultimately, the success of EV-P in daily practice will depend not only on appropriate patient selection but also on the implementation of proactive management and supportive care strategies to ensure safety, treatment continuity, and maximal clinical benefit. This survey provides practical and guideline-informing insights into how clinicians perceive EV-P suitability, but it does not reflect actual treatment behaviors or outcomes.

## Supplementary Material

oyaf333_Supplementary_Data

## Data Availability

All data pertaining to this project are included in the manuscript and the supplementary materials.
