# Supplementary material for: Guiding first-line treatment decisions in advanced urothelial carcinoma: a global survey
Source: Oncologist. 2025 Sep 6;30(10):oyaf333. doi: 10.1093/oncolo/oyaf333 (PMC12558748; doi:10.1093/oncolo/oyaf333)
Supplement: oyaf333_Supplementary_Data [file oyaf333_supplementary_data.docx]

**Title**

**Guiding First-Line Treatment Decisions In Advanced Urothelial Carcinoma: A Global Survey**

**Running head**

**Eligibility conditions to enfortumab vedotin**

Enrique Grande^a,b^, Joaquim Bellmunt^c^, Syed A. Hussain^d^, Mubarak M. Al Mansour^e^, Aristotle Bamias^f^, Philippe Barthélémy^g^, David J. Benjamin^h^, Normand Blais^i^, Maria T. Bourlon^j^, Daniel Castellano^k^, Pongwut Danchaivijitr^l^, Mauricio Fernandez Lazzaro^m^, Patrizia Giannatempo^n^, Félix Guerrero-Ramos^o^, Roberto Iacovelli^p^, Philipp Ivanyi^q^, Eun Hee Jung^r^, Ravindran Kanesvaran^s^, Ray Manneh^t^, Joana C. Marinho^u^, Nobuaki Matsubara^v^, Axel S. Merseburger^w^, Deborah Mukherji^x^, Chandler H. Park^y^, Ben Tran^z^, Karine Martins da Trindade^aa^, Yüksel Ürün^bb^, Ashish M. Kamat^cc^, Alison J. Birtle^dd^

^a^Department of Medical Oncology, MD Anderson Cancer Center Madrid and Universidad Francisco de Vitoria/Facultad de Medicina, Madrid, Spain.

^b^Facultad de Medicina, Universidad Francisco de Vitoria, Pozuelo de Alarcón, Spain

^c^ Department of Internal Medicine, Dana Farber Cancer Institute, Boston, USA.

^d^Division of Clinical Medicine, University of Sheffield, School of Medicine and Population Health, Sheffield, United Kingdom

^e^Princess Noorah Oncology Center, Oncology Department, King Abdulaziz Medical City Ministry of National Guard Health Affairs, Jeddah, Kingdom of Saudi Arabia

^f^2nd Propaedeutic Department of Internal Medicine, National & Kapodistrian University of Athens, Chaidari, Greece

^g^Medical Oncology Department, University Hospital Strasbourg, Strasbourg, France.

^h^Medical Oncology, Hoag Family Cancer Institute, Newport Beach, USA.

^i^Medicine, CHUM, Montreal, Canada

^j^Hemato-Oncology Department, Urologic Oncology Clinic, Instituto Nacional de Ciencias Médicas y Nutrición Salvador Zubirán, Mexico City, Mexico.

^k^Medical Oncology, Hospital Universitario 12 de Octubre, Madrid, Spain.

^l^Department of Medicine, Faculty of Medicine Siriraj Hospital Mahidol University, Bangkok, Thailand

^m^Department of Uro-Oncology, Fundacion COIR, Mendoza, Argentina

^n^Department of Genitourinary Oncology, Fondazione IRCCS Istituto Nazionale dei Tumori, Milan, Italy

^o^Department of Urology, Hospital Universitario 12 de Octubre, Madrid, Spain

^p^Department of Medical and Surgical Sciences, Medical Oncology Unit, Catholic University of Sacro Cure - Fondazione Policlinico Universitario A. Gemelli IRCCS, Rome, Italy

^q^Department of Hematology, Hemostasis, Oncology and Stem Cell Transplantation, Hannover Medical School, Hannover, Germany

^r^Department of Internal Medicine, Division of Hematology and Medical Oncology,
Seoul National University Bundang Hospital, Seongnam-si, Republic of Korea.

^s^Division of Medical Oncology, National Cancer Centre Singapore, Singapore, Singapore

^t^Department of Clinical Oncology, Sociedad de Oncología y Hematología del Cesar, Valledupar, Colombia.

^u^Department of Medical Oncology, Gaia Espinho Local Health Unit, Vila Nova de Gaia,
Portugal

^v^Department of Medical Oncology, National Cancer Center Hospital East, Kashiwa
Japan

^w^Department of Urology, University Hospital Schleswig-Holstein, University Lübeck, Lübeck, Germany

^x^Department of Hematology Oncology, Clemenceau Medical Center Dubai, Dubai, United Arab Emirates

^y^School of Medicine, Norton Cancer Institute/University of Louisville, Louisville, Kentucky, United States

^z^Department of Medical Oncology, Peter MacCallum Cancer Centre, Melbourne, Australia

^aa^Oncologia D’Or, Fortaleza, Brazil.

^bb^Department of Medical Oncology, Ankara University School of Medicine, Ankara, Türkiye.

^cc^Department of Urology, The University of Texas MD Anderson Cancer Center, Houston, United States.

^dd^Department of Oncology, Rosemere Cancer Centre, Lancs Teaching Hospitals; University of Manchester; University of Central Lancashire, Preston, UK

**Corresponding author**

Enrique Grande, MD, PhD

Department of Medical Oncology

MD Anderson Cancer Center Madrid

C. de Arturo Soria, 270

28033 Madrid

Phone: +34917878600

e-mail egrande@oncomadrid.com

**Supplementary material**

**SURVEY FOR ASSESSING EV FEASIBILITY IN DAILY PRACTICE**

Q1. Please mark the most appropriate response according to your daily practice:

- I work in an academic center with ≥1000 beds
- I work in an academic center with 500-999 beds
- I work in an academic center with 200-499 beds
- I work in an academic center with <200 beds
- I work in a community center

Q1. Please mark the most appropriate response according to your daily practice:

- I am Medical/Clinical Oncologist
- I am a urologist
- I am Other (OPEN TEXT)

Q3. Please mark the most appropriate response according to your daily practice:

- I am treating patients with urothelial carcinoma only
- I am treating patients with GU tumors only
- I am treating patients with GU and up to two other solid tumor types
- I treat solid tumor types, including GU tumors

Q4. Please mark the most appropriate response according to your daily practice:

- I participate in the treatment decisions for locally advanced/metastatic bladder cancer within a multidisciplinary tumor board of 1-5 advanced/metastatic UC patients per year.
- I participate in the treatment decisions for locally advanced/metastatic bladder cancer within a multidisciplinary tumor board of 6-10 advanced/metastatic UC patients per year.
- I participate in the treatment decisions for locally advanced/metastatic bladder cancer within a multidisciplinary tumor board of 11-20 advanced/metastatic UC patients per year.
- I participate in the treatment decisions for locally advanced/metastatic bladder cancer within a multidisciplinary tumor board of 21-30 advanced/metastatic UC patients per year.
- I participate in the treatment decisions for locally advanced/metastatic bladder cancer within a multidisciplinary tumor board of >30 advanced/metastatic UC patients per year.
- I participate in the treatment decisions for locally advanced/metastatic bladder cancer within a multidisciplinary tumor board of >50 advanced/metastatic UC patients per year.

Q5. In your opinion, the majority of patients with metastatic UC in your country are treated in:

- Academic environment
- Community-based hospitals

Q6. How many patients have you treated with enfortumab vedotin (EV) so far?

- None
- 1–5
- 6–10
- 11–20
- >20

Q7. Have you participated in a clinical trial involving EV?

- Yes
- No

Q8. In your opinion, as an expert in advanced/metastatic UC, would oncologists treating less than 5 patients per year benefit from having a consensus criterion, based upon expected toxicities, for potential unsuitability for receipt of EV?

- Yes
- No

Q9. Are you considering any of the following criteria in your own clinical practice before using EV? (you can click as many as you wish)

- Galsky’s (criteria for cisplatin eligibility)
- Gupta’s (modified criteria for platinum-ineligibility)
- I don’t use any criteria in my practice (my own clinical judgment).
- Other (free text)

Q10. Which of the following factors are you considering in your own clinical practice to identify patients in whom EV may not be suitable? (you can click as many as you wish)

- Elevated hemoglobin A1c (≥8)
- Grade 2 or higher sensory or motor neuropathy
- Grade 2 or higher corneal or retinal abnormality
- Creatinine clearance of <45 ml/min
- Creatinine clearance of <30 ml/min
- Need for dialysis
- Eastern Cooperative Oncology Group performance status of ≥2
- Age
- Skin disease relevant comorbidity
- Low tumor burden
- Obesity (BMI >35)
- Uncontrolled respiratory disease (risk of pneumonitis)
- Uncontrolled hypertension
- Liver impairment grade 2 or higher (AST/ALT 3 to 5x ULN and/or total bilirubin >1.5 to < 3x ULN) in the absence of liver metastasis
- PDL1 expression
- Nectin-4 expression
- Brain metastasis
- Lymph node-only disease
- NYHA III cardiovascular disease
- Uncontrolled hypertension
- Limited patient social/caregiver support
- Complete pathological response achieved with cisplatin-based chemotherapy in the perioperative treatment
- Frailty of the patient according to standard definitions by geriatric assessment, such as the Charlson score
- Other (free text)

Q11. If you need to define an absolute contraindication for EV, which one of the following would you choose? (you can click as many times as you wish)

- ECOG ≥3
- Elevated hemoglobin A1c (>11)
- Grade ≥2 sensory or motor neuropathy
- Severe corneal or retinal abnormalities
- Creatinine clearance of <30 ml/min
- Need for dialysis
- Liver impairment grade 2 or higher (AST/ALT 3 to 5x ULN and/or total bilirubin >1.5 to < 3x ULN) in the absence of liver metastasis
- Severe skin disease comorbidity (requiring chronic treatment)
- Other (free text)

Q12. If age is relevant for you as a factor conditioning the suitability of a patient to receive EV if the patient otherwise exhibited a performance status of 0-1 with limited comorbidities, what would be the threshold for the use of EV in your practice?

- Age is not a key factor for me
- I am not considering the use of EV in advanced/metastatic UC patients older than 70 y.o.
- I am not considering the use of EV in advanced/metastatic UC patients older than 75 y.o.
- I am not considering the use of EV in advanced/metastatic UC patients older than 80 y.o.
- I am not considering the use of EV in advanced/metastatic UC patients older than 85 y.o.
- I am not considering the use of EV in advanced/metastatic UC patients older than 90 y.o.

Q13. What is your preferred treatment option for a metastatic UC patient ineligible or unsuitable for EV in first line?

- Cisplatin + gemcitabine
- Carboplatin + gemcitabine
- Cisplatin + gemcitabine + nivolumab
- Cisplatin/carboplatin + gemcitabine followed by avelumab in patients who responded
- Single agent atezolizumab
- Single agent pembrolizumab
- Best supportive care

Q14. Do you think that a reliable biomarker is needed in the clinic for the use of EV?

- Yes
- No

Q15. If you responded “YES” to the prior question, which one would you advise to use?

- Nectin-4 expression by IHC in the primary tumor
- Nectin-4 expression by IHC in the metastatic tissue
- Nectin-4 gene amplification
- Other (OPEN Text)

Q16. In terms of the use of EV/P in variant non-urothelial histologies?

- I exclude pure non-urothelial histologies
- I exclude pure non-urothelial histologies if the component is > than 50%
- I do not exclude any type of histology

Q17. Is the cost of EV a barrier to use in clinically eligible patients in your country?

- Yes
- No

Thank you so much for responding to the survey!

**Supplementary table 1**

**Question 5. In your opinion, the majority of patients with metastic UC in your country are treated in**

| **Setting** | n | % |
| --- | --- | --- |
| Community-based hospitals | 104 | 51.7 |
| Academic environment | 97 | 48.3 |

**Supplementary table 2**

**Question 6. How many patients have you treated with enfortumab vedotin (EV) so far?**

| **Number of patients** | n | % |
| --- | --- | --- |
| > 20 | 74 | 36.8 |
| 11-20 | 40 | 19.9 |
| 6-10 | 29 | 14.4 |
| 1-5 | 42 | 20.9 |
| None | 16 | 8.0 |

**Supplementary table 3**

**Q7. Have you participated in a clinical trial involving EV?**

| **Participation** | n | % |
| --- | --- | --- |
| Yes | 109 | 54,2 |
| No | 92 | 45,8 |
| EV, enfortumab vedotin | | |

**Supplementary table 4**

**Q8. In your opinion, as an expert in advanced/metastatic UC, would oncologists treating less than 5 patients per year benefit from having a consensus criterion, based upon expected toxicities, for potential unsuitability for receipt of EV?**

| **Would benefit from having a consensus criterion** | n | % |
| --- | --- | --- |
| Yes | 165 | 82.1 |
| No | 36 | 17.9 |
| EV, enfortumab vedotin; UC, urothelial carcinoma | | |

**Supplementary table 5**

**Q9. Are you considering any of the following criteria in your own clinical practice before using EV?**

| **Criteria** | n | % |
| --- | --- | --- |
| No criteria. Clinical own judgement | 94 | 46.8 |
| Galsky's criteria | 88 | 43.8 |
| Gupta's criteria | 58 | 28.9 |
| EVITA criteria | 10 | 5.0 |
| Comorbidity | 3 | 1.5 |
| Clinical trial with EV | 2 | 1.0 |
| Availability of treatment | 2 | 1.0 |
| M. Committee | 1 | 0.5 |
| Known potential toxicity | 1 | 0.5 |
| Glycemyc and perish neuropathy | 1 | 0.5 |
| Galsky's with a more permissive eGFR cutoff | 1 | 0.5 |
| Economic criteria | 1 | 0.5 |
| Creatinine clearance >50 | 1 | 0.5 |
| Availability & economic access | 1 | 0.5 |
| eGFR, estimated glomerular filtration rate; EV, enfortumab vedotin | | |

**Supplementary table 6**

**Question 10. Which of the following factors are you considering in your own clinical practice to identify patients in whom EV may not be suitable?**

| **Factors** | **n** | **%** |
| --- | --- | --- |
| Grade 2 or higher sensory or motor neuropathy | 170 | 84.6 |
| Eastern Cooperative Oncology Group performance status of ≥2 | 113 | 56.2 |
| Grade 2 or higher corneal or retinal abnormalities | 105 | 52.2 |
| Frailty of the patient according to standard definition by geriatric assessment like Charlson score | 96 | 47.8 |
| Elevated hemoglobin A1C (≥8) | 95 | 47.3 |
| Liver impairment grade 2 or higher (AST/ALT 3 to 5x ULN and/or total bilirubin >1.5 to < 3x ULN) in absence of liver metastasis | 86 | 42.8 |
| Need for dialysis | 78 | 38.8 |
| Skin disease relevant comorbidity | 77 | 38.3 |
| Limited patient social/caregiver support | 73 | 36.3 |
| Creatinine clearance of <30 ml/min | 67 | 33.3 |
| Uncontrolled Respiratory disease (risk of pneumonitis) | 45 | 22.4 |
| NYHA III cardiovascular disease | 45 | 22.4 |
| Age | 25 | 12.4 |
| Creatinine clearance of <45 ml/min | 16 | 8.0 |
| Uncontrolled hypertension | 15 | 7.5 |
| Obesity (BMI >35) | 15 | 7.5 |
| Brain metastasis | 15 | 7.5 |
| Complete pathological response achieved with cisplatin-based chemotherapy in the perioperative treatment | 13 | 6.5 |
| Low tumor burden | 10 | 5.0 |
| Lymph node only disease | 8 | 4.0 |
| Uncontrolled Hypertension | 6 | 3.0 |
| Nectin-4 expression | 3 | 1.5 |
| PDL1 expression | 2 | 1.0 |
| Uncontrolled diabetes mellitus | 1 | 0.5 |
| None of the above. EV is likely better than alternatives in each of these scenarios | 1 | 0.5 |
| None: In the case of EV/Pembrolizumab, since there is no alternative option with comparable efficacy, it is difficult to strictly apply these ineligibility criteria in clinical practice. | 1 | 0.5 |
| Hyperglycemia is not a no-go criteria but requires management | 1 | 0.5 |
| Grade 3 or higher neuropathy affecting QOL/ADL | 1 | 0.5 |
| For older patients, I would consider performing a comprehensive geriatric assessment (Not only frailty) | 1 | 0.5 |
| Availability of treatment | 1 | 0.5 |
| All uncontrolled/severe comorbidities or altered ECOG status might be a no-go for any treatment | 1 | 0.5 |
| ADL, activities of daily life; BMI, body mass index; ECOG PS, Eastern Cooperative Oncology Group Performance Status; EV, enfortumab vedotin; NYHA, New York Heart Association; QOL, quality of life | | |

**Supplementary table 7**

**Question 11. If you need to define an absolute contraindication for EV, which one of the following would you choose?**

| **Absolute contraindication for EV** | **n** | **%** |
| --- | --- | --- |
| Sensory/motor neuropathy grade ≥2 | 129 | 64.2 |
| ECOG-PS ≥3 | 119 | 59.2 |
| Severe corneal/retinal abnormalities | 92 | 45.8 |
| Elevated Hg A1c (>11%) | 91 | 45.3 |
| Severe skin disease | 77 | 38.3 |
| Liver impairment grade ≥2 | 69 | 34.3 |
| Need for dialysis | 66 | 32.8 |
| CrCL<30 ml/min | 43 | 21.4 |
| No contraindications | 2 | 1.0 |
| Sensory/motor neuropathy≥G3 | 2 | 1.0 |
| Neuropathy≥G3 affecting QOL/ADL | 1 | 0.5 |
| Minimal absolute contraindications | 1 | 0.5 |
| Medical history of fall during the last year | 1 | 0.5 |
| I think that the absolute contraindication for EV would be almost the same as platinum-based therapy | 1 | 0.5 |
| G2 neuropathy is a relative contraindication | 1 | 0.5 |
| Case by case basis | 1 | 0.5 |
| ADL, activities of daily life; BMI, body mass index; CrCL, creatinine clearence; ECOG PS, Eastern Cooperative Oncology Group Performance Status; EV, enfortumab vedotin; NYHA, New York Heart Association; QOL, quality of life | | |

**Supplementary table 8**

**Question 12. If age is relevant for you as a factor conditioning the suitability of a patient to receive EV if the patient otherwise exhibited a performance status of 0-1 with limited comorbidities, what would be the threshold for the use of EV in your practice?**

| **Age threshold** | n | % |
| --- | --- | --- |
| Age not key factor | 140 | 69.7 |
| Not-EV adv/mUC pats > 75 y.o. | 1 | 0.5 |
| Not-EV adv/mUC pats > 80 y.o. | 18 | 9.0 |
| Not-EV adv/mUC pats > 85 y.o. | 31 | 15.4 |
| Not-EV adv/mUC pats > 90 y.o. | 11 | 5.5 |

**Supplementary table 9**

**Question 13. What is your preferred treatment option for a metastatic UC patient ineligible or unsuitable for EV in first line?**

| **Treatment** | n | % |
| --- | --- | --- |
| Cisplatin/carboplatin + gemcitabine → avelumab | 107 | 53.2 |
| Cisplatin + gemcitabine + nivolumab | 27 | 13.4 |
| Carboplatin + gemcitabine | 23 | 11.4 |
| Single agent pembrolizumab | 21 | 10.4 |
| Cisplatin + gemcitabine | 10 | 5.0 |
| Best supportive care | 9 | 4.5 |
| Single agent atezolizumab | 4 | 2.0 |

**Supplementary table 10**

**Question 14. Do you think that a reliable biomarker is needed in the clinic for the use of EV?**

| **A reliable biomarker is needed?** | n | % |
| --- | --- | --- |
| No | 125 | 62.2 |
| Yes | 76 | 37.8 |

**Supplementary table 11**

**Question 15. If you responded “YES” to the prior question, which one would you advise to use?**

| **Biomarker** | n | %* |
| --- | --- | --- |
| Nectin-4 expression by IHC in the metastatic tissue | 25 | 32.9 |
| Nectin-4 gene amplification | 24 | 31.6 |
| Nectin-4 expression by IHC in the primary tumor | 10 | 13.2 |
| To be discover | 6 | 7.9 |
| Unknown | 4 | 5.3 |
| Nectin-4 is not best marker | 2 | 2.6 |
| There is no strong biomarker | 1 | 1.3 |
| Nectin-4 and PDl-1 have not been shown to discriminate between responders and non-responders | 1 | 1.3 |
| If patient progressed on nectin-4 drug previously, will be helpful to have IHC on metastatic tissue | 1 | 1.3 |
| Composite of Nectin4 Quantitative score, Her2 quantitative score | 1 | 1.3 |
| Biomarker to exclude rather than biomarker to identify responder | 1 | 1.3 |

*Calculated over those who considered that a reliable biomarker was needed

**Supplementary table 12**

**Question 16. In terms of the use of EV/P in variant non-urothelial histology?**

|  | n | % |
| --- | --- | --- |
| **I don’t exclude any type of histology** | 82 | 40.8 |
| **I exclude pure non-urothelial histologies** | 65 | 32.3 |
| **I exclude pure non-urothelial histologies if the component is greater than 50%** | 54 | 26.9 |

I exclude pure non-urothelial histologies. 32.2%

I exclude pure non-urothelial histologies if the component is greater than 50% 26.9%

Total: 59.2%

**Supplementary table 13**

**Question 17. Is the cost of EV a barrier to use in clinically eligible patients in your country?**

| **Is the cost a barrier?** | n | % |
| --- | --- | --- |
| **Yes** | 119 | 59.2 |
| **No** | 82 | 40.8 |

**Supplementary Figure 1**

**Most frequently (≥5%) proposed factors for evaluating the suitability of using enfortumab vedotin and pembrolizumab as a first-line treatment for patients with advanced urothelial carcinoma
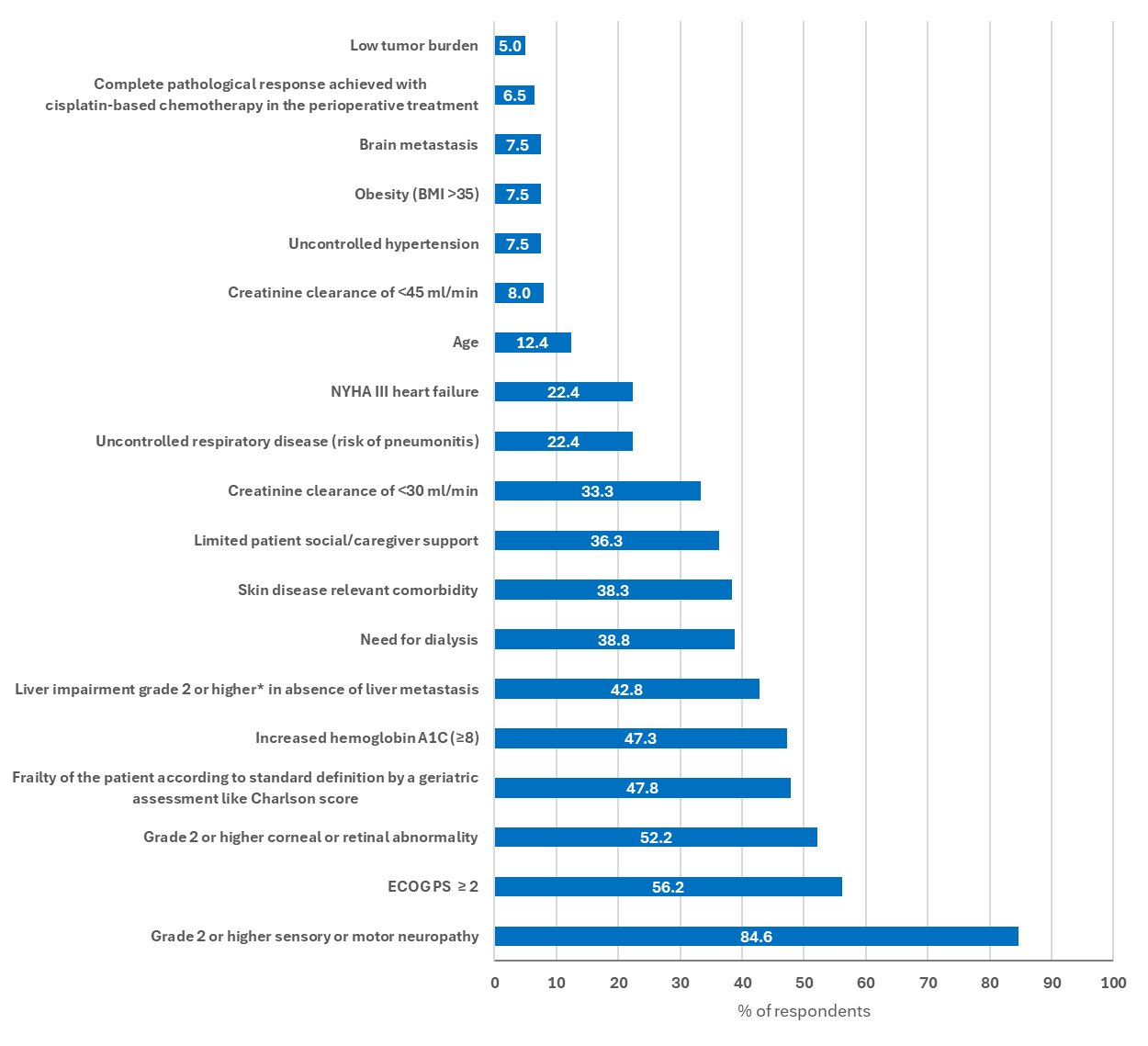
**

*AST/ALT values between 3.0 and 5.0 times the upper limit of normal (ULN) if the baseline was normal and/or bilirubin levels between 1.5 and 3.0 times the ULN if the baseline was normal

BMI, body mass index; ECOG PS, Eastern Cooperative Oncology Group Performance Status; NYHA, New York Heart Association
